# A Prospective Evaluation of Glucagon Stimulation Test Safety in Adults With Chronic Moderate‐to‐Severe Traumatic Brain Injury

**DOI:** 10.1002/edm2.70260

**Published:** 2026-06-15

**Authors:** Corey Snyder, Emily Hillaker, Justin Weppner

**Affiliations:** ^1^ Liberty University College of Osteopathic Medicine Lynchburg Virginia USA; ^2^ Department of Internal Medicine Virginia Tech Carilion School of Medicine Roanoke Virginia USA; ^3^ Edward Via College of Osteopathic Medicine Blacksburg Virginia USA

**Keywords:** glucagon, growth hormone deficiency, traumatic brain injury

## Abstract

**Introduction:**

Growth hormone deficiency (GHD) is a common and clinically significant consequence of moderate‐to‐severe traumatic brain injury (msTBI). The glucagon stimulation test (GST) is widely used to assess growth hormone deficiency; however, the safety of GST has not been systematically evaluated in adults with chronic msTBI.

**Methods:**

This prospective study enrolled 138 adults aged 18–65 years with chronic msTBI (≥ 1‐year post‐injury) from a single academic Brain Injury Center neuroendocrine registry. All participants underwent standardized GST with systematic symptom assessment at baseline and serial intervals over 240 min. Adverse events and symptoms were graded by severity using predefined clinical criteria, and participants were analysed by GHD status and by presence of any adverse event.

**Results:**

GST was well tolerated, with no serious adverse events, test terminations, or prolonged sequelae. The most common adverse effect was nausea (30.4%), with vomiting in 9.4% of participants; antiemetics were required in 16.7% of cases. Female sex was significantly associated with nausea (*p* < 0.001), while age and BMI were not. Neuroglycopenic symptoms occurred in 13% of participants and were mild to moderate; only two individuals (1.5%) required oral glucose rescue, and no severe neuroglycopenia occurred. Headache (12.3%), asthenia (10.1%), and mild symptomatic hypotension (2.9%) were infrequent. Adverse events typically occurred between 60 and 210 min and resolved within the 4 h testing period. There was no significant association between GHD status, peak GH concentration, or adjusted multivariable analysis and the occurrence of adverse events. No seizures occurred, including among participants with post‐traumatic epilepsy.

**Conclusions:**

In adults with chronic msTBI, the glucagon stimulation test demonstrates a favourable safety profile with transient, non‐severe adverse effects and no serious neurological or metabolic complications. These findings support GST as a safe and practical method for evaluating growth hormone deficiency in this population.

## Graphical abstract

The glucagon stimulation test demonstrates a favourable safety profile in adults with chronic moderate‐to‐severe traumatic brain injury, with transient, non‐severe adverse effects and no serious complications.

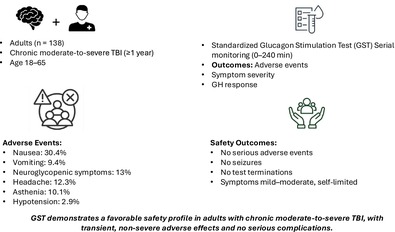

## 1 Introduction

The glucagon stimulation test (GST) is one of the primary evaluation methods used to assess for growth hormone deficiency (GHD) in adult and paediatric populations [1, 2, 3]. The GST provides an indirect, dynamic assessment of the hypothalamic–pituitary axis (HPA). Unlike basal growth hormone measurements, which are limited by the pulsatile nature of GH secretion, it stimulates GH release through metabolic stress mechanisms [1, 4]. The GST is practical, has a favourable safety profile compared to the insulin tolerance test (ITT), and it can be used to evaluate both growth hormone and adrenocorticotropic hormone (ACTH) secretion [1, 2, 3, 4, 5]. Although serum insulin‐like growth factor‐1 (IGF‐1) may be utilized as an initial screening tool in the evaluation of suspected GHD, it cannot be used alone to establish the diagnosis [6]. Untreated GHD can adversely affect bone health, metabolism, body composition, and quality of life [1, 2, 3]. Traumatic brain injury (TBI) is both prevalent and a leading cause of chronic neurological and endocrine dysfunction, most commonly GHD as well as other hypothalamic–pituitary deficits [7, 8, 9, 10]. Up to 50% of individuals experience some form of pituitary hormone deficiency following a moderate‐to‐severe TBI (msTBI) [7, 9]. Evaluation and recognition of GHD is imperative in the post‐TBI population, as treatment with hormone replacement therapy can significantly improve cognition, energy, and quality of life [7, 10].

The GST has been extensively studied in adult and paediatric cases of idiopathic or structural pituitary dysfunction; however, data on the safety profile of the GST in TBI populations are scarce. Reported adverse effects of the GST in the general population include nausea, vomiting, diaphoresis, hypotension and hypoglycemia‐related neurological symptoms such as dizziness or syncope [3, 5, 11]. In patients with TBI, the physiological response to glucagon and the risk of hypoglycemia may be altered, emphasizing the need for further investigation and highlighting an important gap in the existing literature [4, 8, 11]. Structural and functional injury involving the HPA and autonomic nervous system may be associated with dysregulated neuroendocrine stress responses and impaired glucose regulation following TBI [9, 12, 13]. Additionally, abnormalities in cerebral glucose metabolism and neuroendocrine/autonomic dysfunction after TBI may increase susceptibility to neuroglycopenic or autonomic symptoms during endocrine testing [12, 14]. There is potential for disrupted metabolic regulation, compromised counterregulatory mechanisms, and increased risk of adverse events after brain injury. Therefore, it is important to assess whether the safety profile of GST observed in non‐TBI populations can be generalized to patients with TBI, especially in light of the clinical implications of GHD in the TBI population [12, 14]. The current study aims to assess the safety profile and characterize the associated symptoms and adverse events of the GST in patients with a history of TBI. To our knowledge, this is the first study to report GST‐associated symptoms and adverse events in an adult msTBI population.

## 2 Methods

### 2.1 Participants

This prospective study enrolled 138 individuals between 18 and 65 years of age with moderate‐to‐severe TBI (msTBI) into a neuroendocrine registry at a single academic Brain Injury Center. The study protocol received approval from the institution's Institutional Review Board (IRB) (IRB‐24‐1930), and all participants provided written informed consent prior to enrolment. Eligibility was defined by at least one of the following criteria within the first 24 h post‐injury: a best Glasgow Coma Scale score between 3 and 12, loss of consciousness lasting longer than 30 min, or post‐traumatic amnesia extending beyond 24 h. Exclusion criteria were as follows: presence of three or more hormonal deficiencies with an IGF‐1 z‐score less than −2, inability to provide informed consent due to significant cognitive impairment, current pregnancy, recent pituitary surgery (within 6 weeks), active acromegaly or pheochromocytoma, liver transaminases exceeding three times the upper normal limit, serum creatinine above 2 mg/dL, active malignancy within the preceding 5 years, uncontrolled hypertension, severe acute illness, prior growth hormone therapy, malnutrition, or fasting blood glucose greater than 180 mg/dL.

Assessment for GHD was performed at least one year post‐injury to allow for spontaneous recovery of pituitary function. Prior to dynamic testing, all subjects underwent a comprehensive neuroendocrine workup, including evaluation of thyroid function (TSH, T4), gonadotropins (FSH, LH), sex hormones (testosterone or oestrogen as appropriate), prolactin, cortisol, IGF‐1, and a full metabolic panel. Any detected endocrine abnormalities were treated and stabilized before GST administration. Adverse events occurring during the GST were systematically documented. Throughout the GST, adverse effects were prospectively and systematically recorded to ensure comprehensive safety monitoring. Side effects were assessed at baseline (0 min) prior to glucagon administration and subsequently at 30, 60, 90, 120, 150, 180, 210, and 240 min. If a participant reported a side effect between scheduled evaluations, it was documented at the time of reporting and assigned to the nearest scheduled reporting interval for analysis. At each time point, participants were queried regarding the presence, onset, severity, and progression of symptoms, allowing investigators to distinguish baseline findings from test‐related effects and to characterize the temporal pattern of adverse events over the full duration of the GST.

### 2.2 Symptom Definitions

Symptoms were graded clinically as mild, moderate, or severe based on participant subjective reporting, functional impact, and need for intervention. Symptoms were graded clinically as mild when they were subjectively reported with minimal functional impact and no need for intervention, moderate when they caused noticeable functional limitation and required some intervention from medical staff, and severe when they resulted in significant functional impairment, such as syncope from hypotension and necessitated urgent medical intervention. Neuroglycopenic symptoms were defined as a glucose level of < 70 mg/dL and patient‐reported symptoms consistent with central nervous system glucose deprivation, including shakiness, sweating, hunger, anxiety, dizziness, or irritability. Mild neuroglycopenia was defined as symptoms requiring no intervention. Moderate neuroglycopenia was defined as symptoms requiring intervention but not meeting criteria for severe neuroglycopenia. Severe neuroglycopenia was defined as confusion, disorientation, difficulty speaking, or loss of consciousness. Oral glucose rescue was administered for persistent or worsening neuroglycopenic symptoms at the discretion of the supervising clinician. No predefined glucose threshold alone mandated test termination; rather, testing was discontinued only in the event of clinically significant or progressive symptoms at the discretion of the attending physician, consistent with symptom‐based safety monitoring principles. Symptomatic hypotension was defined as a ≥ 20 mmHg systolic or ≥ 10 mmHg diastolic drop from baseline, or a systolic BP < 90 mmHg, accompanied by symptoms such as dizziness, lightheadedness, presyncope/syncope, visual disturbance, or cognitive slowing. Other adverse effects, including nausea, vomiting, headache, and asthenia, were documented throughout the testing period.

### 2.3 GST

The GST was conducted using a standardized approach to provoke GH release. Participants were required to fast overnight for 8–10 h, during which only water was allowed. Usual morning medications could be taken with water, except steroids and diabetes medications, which were held on the day of the GST. Upon arrival, each participant's weight was measured to determine the correct glucagon dosage. An intravenous cannula was inserted into a forearm vein to enable repeated blood draws. Glucagon was given intramuscularly, with a dose of 1.0 mg for those weighing 90 kg or less, and 1.5 mg for those above 90 kg. Blood samples and vital signs were obtained at baseline (0 min) and then at 30, 60, 90, 120, 150, 180, 210, and 240 min after glucagon injection. These samples were analysed for both serum GH and blood glucose levels. Interpretation followed established BMI‐appropriate GH cutoffs for the GST: a peak GH cutoff of 3 μg/L for normal‐weight (BMI < 25 kg/m^2^) and overweight (BMI 25–30 kg/m^2^) patients with a high pretest probability of GHD, and a lower cutoff of 1 μg/L for obese (BMI > 30 kg/m^2^) and overweight (BMI 25–30 kg/m^2^) patients with a low pretest probability, given the reduced glucagon‐induced GH response associated with increasing BMI [3].

### 2.4 Statistical Analysis

This investigation represents a secondary analysis of data derived from an ongoing prospective neuroendocrine registry study. As the parent study was not originally designed or powered to evaluate adverse event frequency during the GST, a priori power calculations specific to the current safety outcomes were not performed. Given the exploratory and safety‐focused nature of this secondary analysis, results are interpreted descriptively, with inferential statistics used to identify potential associations rather than to establish definitive causal relationships. Statistical analyses were performed to characterize the frequency, type, and predictors of adverse events associated with the GST in adults with TBI. Descriptive statistics were used to summarize demographic and clinical characteristics, with continuous variables reported as mean ± standard deviation, as appropriate, and categorical variables reported as counts and percentages. Participants were grouped in two ways: (1) by GHD status (GHD vs. GH‐sufficient) based on peak GH cut‐points, and (2) by the presence or absence of any GST‐related adverse event. Normality of continuous variables was assessed using visual inspection of histograms and the Shapiro–Wilk test. Continuous variables were analysed using parametric tests when normality assumptions were met. Between‐group comparisons were conducted using independent *t*‐tests for continuous variables and chi‐square tests for categorical variables. The proportion of participants experiencing any AE, as well as specific symptoms (nausea, vomiting, neuroglycopenic symptoms, headache, asthenia, and hypotension), was compared between GHD and GH‐sufficient groups. Risk ratios (RRs) with 95% confidence intervals were calculated to estimate the association between GHD status and the occurrence of any adverse event as well as individual symptoms (nausea, vomiting, neuroglycopenic symptoms, headache, asthenia, and hypotension). Mean peak GH concentrations were compared between participants with and without AEs. To further evaluate predictors of adverse events, multivariable logistic regression analysis was performed with occurrence of any AE as the dependent variable and peak GH concentration as the primary independent variable, adjusting for age, sex, and BMI. Adjusted odds ratios with 95% confidence intervals were calculated. The timing and resolution of adverse events across GST time points were analysed descriptively. All tests were two‐sided, with statistical significance defined as *p* < 0.05. Statistical analyses were conducted using SPSS 29.

## 3 Results

Demographic and injury characteristics of the study cohort are summarized in Table 1. A total of 27 out of 138 participants (19.6% (95% CI 13.7%–27.1%)) tested positive for GHD. In the cohort of patients with msTBI who underwent the GST, the most frequently reported side effect was nausea, occurring in 30.4% (95% CI 22.9%–38.8%) of cases, with vomiting observed in 9.4% (95% CI 5.4%–15.7%) of patients. Antiemetic medications were required in 16.7% of symptomatic patients with nausea. Female sex was associated with a higher incidence of nausea, with 53.6% of females experiencing nausea compared with 24.5% of males (*p* = 0.006), whereas age (34 ± 13.3 vs. 35 ± 14 years, *p* = 0.68) and BMI (25 ± 4.8 vs. 26 ± 4.3 kg/m^2^, *p* = 0.19) did not differ significantly between those with and without nausea. Neuroglycopenic symptoms occurred in 13% (95% CI 8.3%–19.8%) of patients and were mild to moderate in severity, with two participants (1.5% (95% CI 0.4%–5.2%)) requiring oral glucose rescue. No participants reported severe symptoms such as confusion, disorientation, difficulty speaking, or loss of consciousness. Additional side effects included headache (12.3% (95% CI 7.7%–19.1%)), asthenia (10.1% (95% CI 6.0%–16.6%)), and mild symptomatic hypotension (2.9% (95% CI 1.0%–8.1%)) (Tables 2 and 3).

**TABLE 1:** Demographic and injury characteristics of the study cohort (*N* = 138).

Characteristic	Value
Age, years	35 ± 14.1
Sex, *n* (%)	
Male	110 (79.7)
Female	28 (20.3)
Race, *n* (%)	
White	123 (89.1)
Black	11 (8.0)
American Indian or Alaskan Native	1 (0.7)
Asian	3 (2.2)
Body mass index, kg/m^2^	26 ± 4.4
TBI severity, *n* (%)	
Severe	112 (81.2)
Moderate	26 (18.8)
Mechanism of injury, *n* (%)	
Motor vehicle collision	72 (52.2)
Falls	26 (18.8)
Blunt (struck head against object)	26 (18.8)
Assaults	10 (7.2)
Penetrating head injury	2 (1.4)
Blast head injury	2 (1.4)

*Note:* Results are reported as mean ± standard deviation or as number (percentage).

**TABLE 2:** Frequency and timing of adverse effects during the glucagon stimulation test.

Time points	Nausea	Vomiting	Headache	Asthenia	Hypotension	Neuroglycopenia
	42 (30.4%)	13 (9.4%)	17 (12.3%)	14 (10.1%)	4 (2.9%)	18 (13%)
Baseline	0 (0%)	0 (0%)	0 (0%)	0 (0%)	0 (0%)	0 (0%)
30 min	0 (0%)	0 (0%)	0 (0%)	0 (0%)	0 (0%)	0 (0%)
60 min	2 (4.8%)	0 (0%)	0 (0%)	1 (7.1%)	0 (0%)	0 (0%)
90 min	3 (7.1%)	0 (0%)	1 (5.9%)	1 (7.1%)	0 (0%)	1 (5.6%)
120 min	18 (42.9%)	1 (7.7%)	3 (17.6%)	1 (7.1%)	0 (0%)	4 (22.2%)
150 min	11 (26.1%)	5 (38.5%)	5 (29.4%)	2 (14.3%)	1 (25%)	5 (27.8%)
180 min	6 (14.3%)	3 (23.1%)	6 (35.3%)	7 (50%)	2 (50%)	7 (38.9%)
210 min	2 (4.8%)	4 (30.8%)	2 (11.8%)	2 (14.3%)	1 (25%)	1 (5.6%)
240 min	0 (0%)	0 (0%)	0 (0%)	0 (0%)	0 (0%)	0 (0%)

*Note:* Values are presented as *n* (%) of participants experiencing each symptom at the specified time point. Percentages reflect the proportion of all reported events for each adverse effect across the test duration. No adverse effects were observed at baseline, 30 min, or 240 min.

**TABLE 3:** Heat map of adverse effects during glucagon stimulation test.

Time points	Nausea	Vomiting	Headache	Asthenia	Hypotension	Neuroglycopenia
	42 (30.4%)	13 (9.4%)	17 (12.3%)	14 (10.1%)	4 (2.9%)	18 (13%)
Baseline	0	0	0	0	0	0
30 min	0	0	0	0	0	0
60 min	2	0	0	1	0	0
90 min	3	0	1	1	0	1
120 min	18	1	3	1	0	4
150 min	11	5	5	2	1	5
180 min	6	3	6	7	2	7
210 min	2	4	2	2	1	1
240 min	0	0	0	0	0	0

*Note:* Cell values represent the number of participants reporting each symptom at each time interval. Column headers indicate the total number and percentage of participants who experienced each symptom at any time during the GST. Darker color intensity on the heat map corresponds to higher symptom frequency. No adverse effects were observed at baseline, 30 min, or 240 min.

For all other documented symptoms, age, sex, BMI, and TBI severity did not differ significantly between participants with and without symptoms (all *p* > 0.05). The onset of these symptoms typically occurred between 60 and 210 min following glucagon administration, corresponding to the period of peak growth hormone responses. Importantly, all side effects resolved spontaneously within 4 h of test initiation, and no serious adverse events or prolonged sequelae were observed. No GST was terminated prematurely because of adverse events or symptoms during the study period. Patients with msTBI are known to have an increased risk of post‐traumatic epilepsy; in this cohort, 13% carried a diagnosis of post‐traumatic epilepsy, with no seizures occurring during the GST.

There was no significant association between GHD status and the occurrence of adverse effects during the GST. The proportion of participants experiencing any adverse event did not differ between those diagnosed with GHD and those who were GH‐sufficient (*p* = 0.41). Similarly, the frequencies of nausea (*p* = 0.52), vomiting (*p* = 0.63), neuroglycopenic symptoms (*p* = 0.28), headache (*p* = 0.47), asthenia (*p* = 0.35), and hypotension (*p* = 0.59) were comparable between groups. The risk of experiencing any adverse event was similar between participants with GHD and those who were GH‐sufficient (RR 1.28, 95% CI 0.74–2.23). Likewise, the risks of individual adverse events were comparable between groups, including nausea (RR 1.28, 95% CI 0.74–2.23), vomiting (RR 1.23, 95% CI 0.37–4.12), neuroglycopenic symptoms (RR 1.17, 95% CI 0.43–3.22), headache (RR 1.27, 95% CI 0.45–3.54), asthenia (RR 1.12, 95% CI 0.34–3.70), and hypotension (RR 1.37, 95% CI 0.15–12.7), with all confidence intervals crossing 1.0 and consistent with no clinically meaningful difference. When analysed as a continuous variable, mean peak GH levels did not differ significantly between participants who experienced any adverse event and those who did not (*p* = 0.33). Logistic regression analysis likewise demonstrated that peak GH concentration was not a significant predictor of adverse events after adjustment for age, sex, and BMI (*p* = 0.62). The magnitude of GH response during the GST was not associated with test‐related side effects in adults with chronic msTBI.

## 4 Discussion

The primary goal of this study was to evaluate the safety profile of the GST in adults with a history of msTBI. In this prospective study, the GST displayed a favourable safety profile without serious adverse events. Notably, no test was terminated prematurely, and all patient‐reported symptoms resolved completely within the testing window. In this cohort, the most reported adverse effect was nausea (30.4%), with vomiting occurring in 9.4%. This is consistent with prior GST studies in non‐TBI adults, which report 15%–44% of patients experiencing nausea and 2%–10% experiencing vomiting, suggesting that the known adverse effects of this test are generalizable to the TBI population [2, 3, 4]. Additionally, this study was consistent with prior research in non‐TBI populations demonstrating a higher incidence of nausea among females, further reinforcing similar observations reported in previous GST cohorts [3].

While neuroglycopenic symptoms were reported, they were infrequent, occurring in only 13% of participants. Of these, only 1.5% required oral glucose rescue, compared with 7.6% reported in prior literature among non‐TBI populations [3]. In this study, there were no reported episodes of severe neuroglycopenia, loss of consciousness, or altered mental status. Impaired glucose regulation and autonomic dysfunction are concerns for patients following TBI [6, 7, 8, 12, 13, 14]. Interestingly, no clinically significant hypoglycemia occurred, suggesting preserved physiological safety during GST. Importantly, seizures were not observed or reported during GST administration, including within the subset of patients with a history of post‐traumatic epilepsy [6, 7, 9]. The fact that no patients in this study developed a seizure during GST administration is reassuring regarding the neurological safety of GST in a population with an elevated baseline seizure risk. Temporal clustering of symptoms between 60 and 210 min following administration of glucagon aligns with the expected peak growth hormone responses, which supports a physiological response rather than TBI‐specific predisposition to these adverse events [3, 4, 5]. All symptoms resolved spontaneously within 4 h, supporting the transient nature of GST‐associated adverse events in this population [1, 2, 3, 4]. No clinical variables, including age, body mass index, or TBI severity, were associated with increased adverse events, and GST intolerance appears difficult to predict based on baseline clinical characteristics alone in the msTBI population [3, 4, 5]. This is similar to the non‐TBI population where GST intolerance is difficult to predict based on clinical characteristics. This study highlights that GST is well tolerated broadly across diverse presentations in adults following msTBI.

Prior studies have not demonstrated a clear relationship between the magnitude of growth hormone response and the occurrence or severity of symptoms during the GST [5]. Similarly, in our cohort of adults with chronic msTBI, adverse events were not associated with categorical GHD status or with peak GH levels analysed as a continuous variable, even after adjustment for age, sex, and BMI. These findings extend the existing safety data by demonstrating that the degree of GH deficiency does not appear to influence the likelihood of test‐related side effects, further supporting the GST as a safe dynamic diagnostic test to assess for GHD.

### 4.1 Limitations

The main strength of this study is the prospective and systematic collection of data during the testing period of the GST in a cohort of adults with msTBI. However, there are several limitations that should be acknowledged. Primarily, the study represents a secondary, exploratory safety analysis using data derived from a neuroendocrine registry at a single academic Brain Injury Center, which may limit the generalizability of the findings to clinical settings that use different GST procedures or monitoring methods. The study cohort was limited to adults with msTBI evaluated at least one year following their injury. As a result, these findings may not apply to patients with mild TBI or other individuals presenting for endocrine evaluation in the acute post‐injury period, when physiological susceptibilities to adverse events may vary. The absence of a concurrently tested non‐TBI control group does not allow for a direct comparison of present symptoms and frequency of adverse events between patients with msTBI and non‐TBI populations undergoing GST. Lastly, several adverse events were assessed by reports from patients based on their symptoms. While this does reflect clinical practice, it does leave the possibility for reporting bias and may lead to under recognition of physiological effects that are not reported.

## 5 Conclusions

In adults with chronic msTBI, the GST demonstrated a favourable safety profile with no serious adverse events, no test terminations, and complete symptom resolution within the testing period. Adverse effects were not severe, with the most commonly reported being nausea, and adverse effects were consistent with those reported in non‐TBI populations. Importantly, no severe hypoglycemia, seizures, or neurologic complications occurred, including among patients with post‐traumatic epilepsy. These findings support the hypothesis that GST is a safe and practical method for evaluating GHD in adults with chronic msTBI.

## Author Contributions

**Emily Hillaker:** writing – review and editing, data curation. **Justin Weppner:** conceptualization, investigation, writing – original draft, methodology, validation, visualization, writing – review and editing, formal analysis, project administration, data curation, supervision. **Corey Snyder:** conceptualization, data curation, writing – original draft.

## Funding

The authors have nothing to report.

## Ethics Statement

The study protocol was approved by the Carilion Clinic Institutional Review Board (IRB‐24‐1930).

## Consent

Written informed consent was obtained from all participants prior to enrolment.

## Conflicts of Interest

The authors declare no conflicts of interest.

## Data Availability

The data that support the findings of this study are available from the corresponding author upon reasonable request.
